# Deciphering morphological and biochemical responses of *Salvia leriifolia* to seed cold plasma treatment, priming, and foliar spraying with nano-salicylic acid

**DOI:** 10.1038/s41598-023-45823-8

**Published:** 2023-10-31

**Authors:** Seyedeh Parisa Ghodsimaab, Ziba Ghasimi Hagh, Hassan Makarian, Manoochehr Gholipoor

**Affiliations:** 1 Department of Agronomy and Plant Breeding, Faculty of Agriculture, Shahrood University of Technology, Shahrood, 3619995161 Iran; 2 Department of Horticulture Science and Plant Protection, Faculty of Agriculture, Shahrood University of Technology, Shahrood, 3619995161 Iran

**Keywords:** Plant hormones, Plant physiology, Secondary metabolism

## Abstract

The pretreatment of seeds with cold plasma (CP) (0 and 100 w for 240 s), and salicylic acid priming (SA) (0 and 2 mM normal and nano form), and foliar spraying of SA at the six-leaf stage (0 and 2 mM normal and nano form) of *Salvia leriifolia* plants in field condition was studied. Compared to the control plants of *S. leriifolia*, the results showed that CP + both forms of SA priming + nano-SA spraying increased plant height, leaf length, plant dry weight, total phenol, and the activities of phenylalanine ammonia-lyase (PAL) and tyrosine ammonia-lyase (TAL) enzymes. The chlorophyll a and b contents in all treated plants remained either unchanged or decreased when compared to the control. The highest PAL activity was obtained in CP-free + hydro-priming + nano-SA foliar spraying. The highest content of caffeic acid was achieved in CP + SA priming + SA foliar spraying in the leaf. The maximum contents of rosmarinic and salvianolic acid were obtained in the control plants. In conclusion, CP and nano-SA can increase PAL and TAL activity and total phenol accumulation in *S. leriifolia* plants, but not rosmarinic and salvianolic acid contents. Other phenolic compound enzymes and their production require further study.

## Introduction

*Salvia leriifolia* Benth is a dicotyledonous perennial medicinal plant belonging to the Lamiaceae family (formerly called Labiatae). It features with grey-green aromatic foliage and fragrant purple-white flowers and is native to the Semnan and Khorasan provinces of Iran^1^. The secondary metabolites of *S. leriifolia* have demonstrated significant utility in the treatment of conditions such as thrombosis, hypertension, convulsant, ischemia, inflammation, cancer, and Alzheimer's disease, as indicated by previous research on the medicinal and therapeutic properties of this plant. Additionally, *S. leriifolia* has exhibited antivirus, antibacterial, antifibrotic, and cardioprotective properties^2–5^.

**Figure 1:**
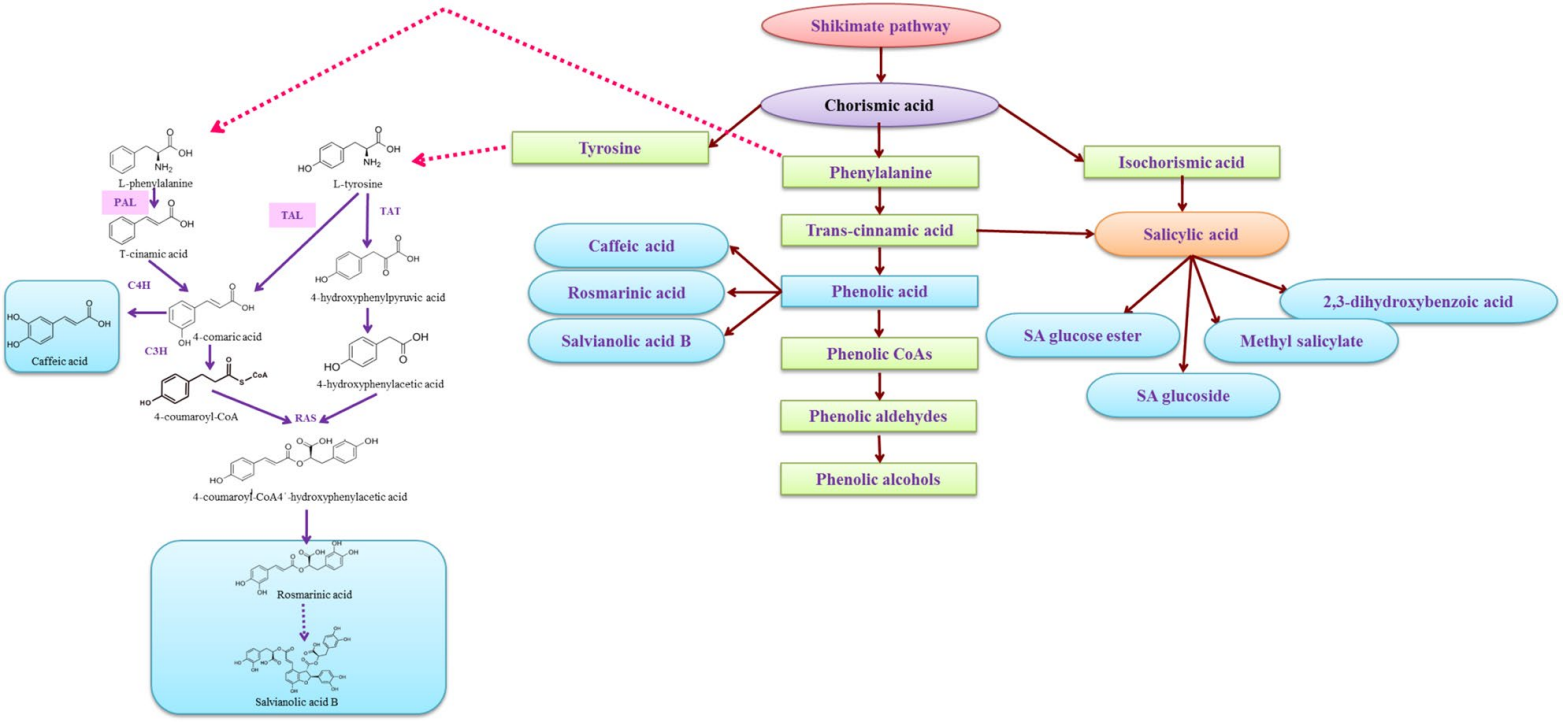
Biosynthesis pathway of phenolic acids and salicylic acid from chorismic acid and phenylalanine.

The identified medicinal properties of plants in the Salvia genus are related to their phytochemical content, including terpenoids, saponins, flavonoids, tannins and alkaloids^6,7^. Modarres et al.^7^ introduced salvianolic acid B, caffeic acid and rosmarinic acid as the main phenolic compounds in *S. leriifolia*. The transformation of L-tyrosine and phenylalanine into these secondary metabolites in the phenylpropanoid pathway begins with the intermediation of two enzymes, tyrosine aminotransferase (TAT) and phenylalanine ammonia-lyase (PAL) (Fig. 1). Throughout the various stages of plant development, from seed to harvest, a wide range of elicitors, assisted by signaling molecules, can stimulate the induction of secondary metabolites in plants^8^. Previous research on the biosynthesis and content of phenolic compounds in *S. leriifolia*, particularly in callus, plantlets, and suspension cultures, has demonstrated that certain elicitors, including polyethylene glycol^9^, methyl jasmonate (MeJA), and salicylic acid (SA)^10^, as well as factors like light^11^, and ultraviolet-A or B (UV-A or B) radiation^12^), exert a significant influence on their biosynthesis.

Recent advancements in agriculture have introduced nanotechnology and cold plasma (CP) therapy as the latest technologies with reported beneficial effects on various aspects of plant physiology^13^. CP can enhance seed permeabilityto water, and gases through physical and chemical changes to the seed cover^14^. It also increases the activity of enzymes involved in germination, and plant growth^15^, and influences the biosynthesis of secondary metabolites^16^. Additionally, CP reduces seed pathogens that damage seed germination^17^. The effectiveness of CP on plants depends on factors such as the types of electrical discharge, combinations of ground gas and pressure, and electrical or electromagnetic sources^18^. Nanoparticles, due to their small size, can easily pass through cell wall pores and reach the plasma membraneallowing them to participate in the cellular activities^19^. Salicylic acid (SA), a plant hormone, is well-known for its crucial roles in basal defense and amplification of local immune responses^20^. SA collaborates with other plant hormones to regulate processes such as photosynthesis, respiration, seed germination, thermogenesis, senescence, vegetative growth, flowering, and mediation in maintaining a relatively stable cellular redox in plants^21–25^. Based on previous studies, SA can trigger the biosynthesis and accumulation of metabolites in plants^26–28^. Thus, the positive effect of this elicitor have been reported on the accumulation of total leaf phenols in *Aloysia citrodora*^29^, the content of rosemarinic acid, caffeic acid, and salvianolic acid in *S. leriifolia*^10^, the content of polyphenol and carotenoid in *Lavandula angustifolia*^28^, and the content of flavonolignans in *Silybum marianum*^26^. Changing the size of SA from normal to nano size revealed that foliar spraying of Nano-SA was more effective than SA on the height, fresh and dry weight of shoots and roots, and the content of anthocyanins in *Lycopersicon esculentum* Mill and *Isatis cappadocica* plants^30,31^.

Uniform seed germination and normal emergence are crucial factors in the initial and subsequent vegetative growth of plants, and the biosynthesis of secondary metabolites. Therefore, to enhance the biosynthesis of secondary metabolites in salvia, the effect of CP and nano-SA during the germination stage, in combination with foliar spraying of nano-SA at the true six-leaf stage, were studied to investigate their bio-physiological responses.

## Results

### The particle size of nano-salicylic acid

**Figure 2:**
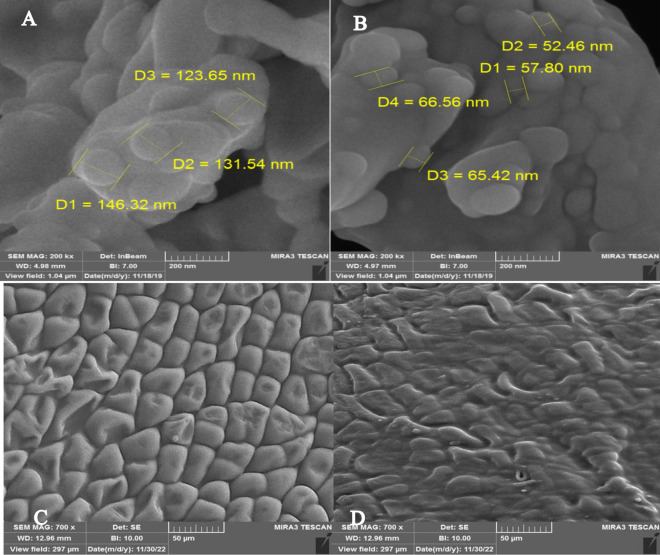
FESEM photograph of salicylic acid (SA) and *Salvia leriifolia* seed. (**A**) Salicylic acid, (**B**) salicylic acid nanoparticles (nano-SA), (**C**) uncoated *S. leriifolia* seed (cold plasma-free) and (**D**) uncoated *S. leriifolia* seed exposed to cold plasma (100 W, 240 S).

Comparing Field Emission Scanning Electron Microscopy (FESEM) images confirmed a distinct difference in morphology between normal SA and nano-SA. Nano-SA exhibited proper dispersion, consisting of particles averaging < 100 nm, whereas normal SA particles were larger, exceeding 100 nm. FESEM images showed that nano-SA had an oval morphology with an average size ranging from 52 and 66 nm (Fig. 2A,B).

### Seed surface properties by SEM

The morphological changes of *S. leriifolia* seed surface caused by FR-cold plasma are depicted in Fig. 2C and D. The SEM images reveal that RF-CP, in combination with SA priming, had a significant etching effect on the surfaces of un-coated seeds. While the surface of CP-free seeds exhibited a regular mesh-like structure, this structure was notably disrupted after four minutes of RF-CP exposure. This morphological alteration increased the permeability of the tissue.

### Plant height

**Figure 3:**
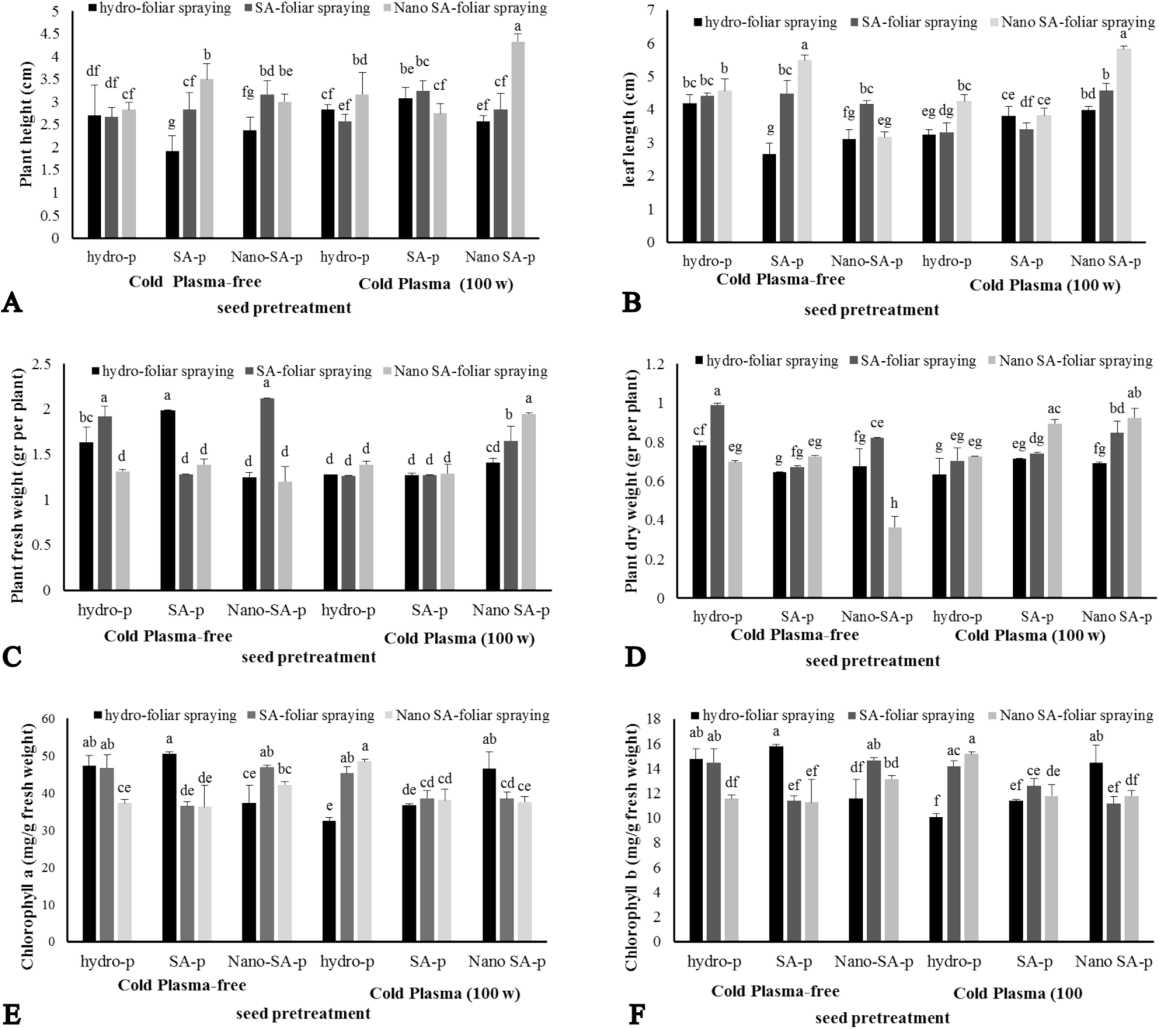
The effect of seed treatment with cold plasma and nano-salicylic acid and foliar spraying of nano-salicylic acid on plant height (**A**), leaf length (**B**), fresh weight (**C**), dry weight (**D**) and chlorophyll a (**E**) and b (**F**) of *S. leriifolia* plant. Values represent mean ± S.E. Different letters indicate significant differences at the 1% level according to the LSD test.

Enhanced plant height was observed through the application of CP, seed priming, and foliar spraying of SA in both normal and nano forms. The tallest *S. leriifolia* plants, reaching a height of 4.33 cm, were obtained when using CP + nano-SA priming + nano-SA foliar spraying, representing a significant (P ≤ 0.01) difference compared to other treatments (Fig. 3A). Conversely, the shortest plant height, measuring 1.92 cm, was obtained in the CP-free + SA priming + hydro-foliar spraying treatment, resulting in a twofold decrease compared to the maximum height (Fig. 3A).

### Leaf length

The results of the three-way interactions between CP, SA priming, and SA foliar spraying on leaf length showed that, similar to plant height (P ≤ 0.01), the highest leaf length was recorded in two treatments : CP-free + SA priming + nano-SA foliar spraying (5.5 cm) and CP + nano-SA priming + nano-SA foliar spraying (5.83 cm) (Fig. 3B).

### Fresh and dry weight of plants

The results regarding the effect of CP, SA priming, and SA foliar spraying on the fresh and dry weight of *S. leriifolia* plants are presented in Fig. 3C and D, respectively. The obtained results showed, that the maximum weight was observed in treatment combinations, including CP-free + nano-SA priming + SA foliar spraying (P ≤ 0.01). Interestingly, the fresh weight of *S. leriifolia* plants did not significantly differ in most of the treatment combinations. For example, CP + hydro-priming and CP + normal SA-priming did not show significant differences in all foliar sprayings. However, CP + nano-SA priming had a positive and significant effect on fresh weight. In contrast to the results obtained for fresh weight, using CP + priming and foliar spraying of both forms of SA increased the dry weight compared to hydro priming, with nano-SA showing a more significant increase compared to normal SA. The treatment combinations, including CP-free + hydro-priming + SA foliar spraying and CP + nano-SA priming + nano-SA foliar spraying, displayed the maximum dry weight (0.987 and 0.925 g) compared to the control (0.783 g) (Fig. 3D).

### Chlorophyll a and b

The chlorophyll a and b data indicated that the chlorophyll a and b content of *S. leriifolia* plants in response to the treatments was comparable (P ≤ 0.01) (Fig. 3E,F). These chlorophylls were found in the greatest concentration in the control plants. In some treatment combinations, the content of chlorophyll a and b was reduced compared to the control. It appears that the combination of CP + priming + foliar spraying has resulted in a greater reduction reduced more compared to the CP-free treatment (Fig. 3E,F).

### Phenylalanine ammonia-lyase and tyrosine ammonia-lyase activity

**Figure 4:**
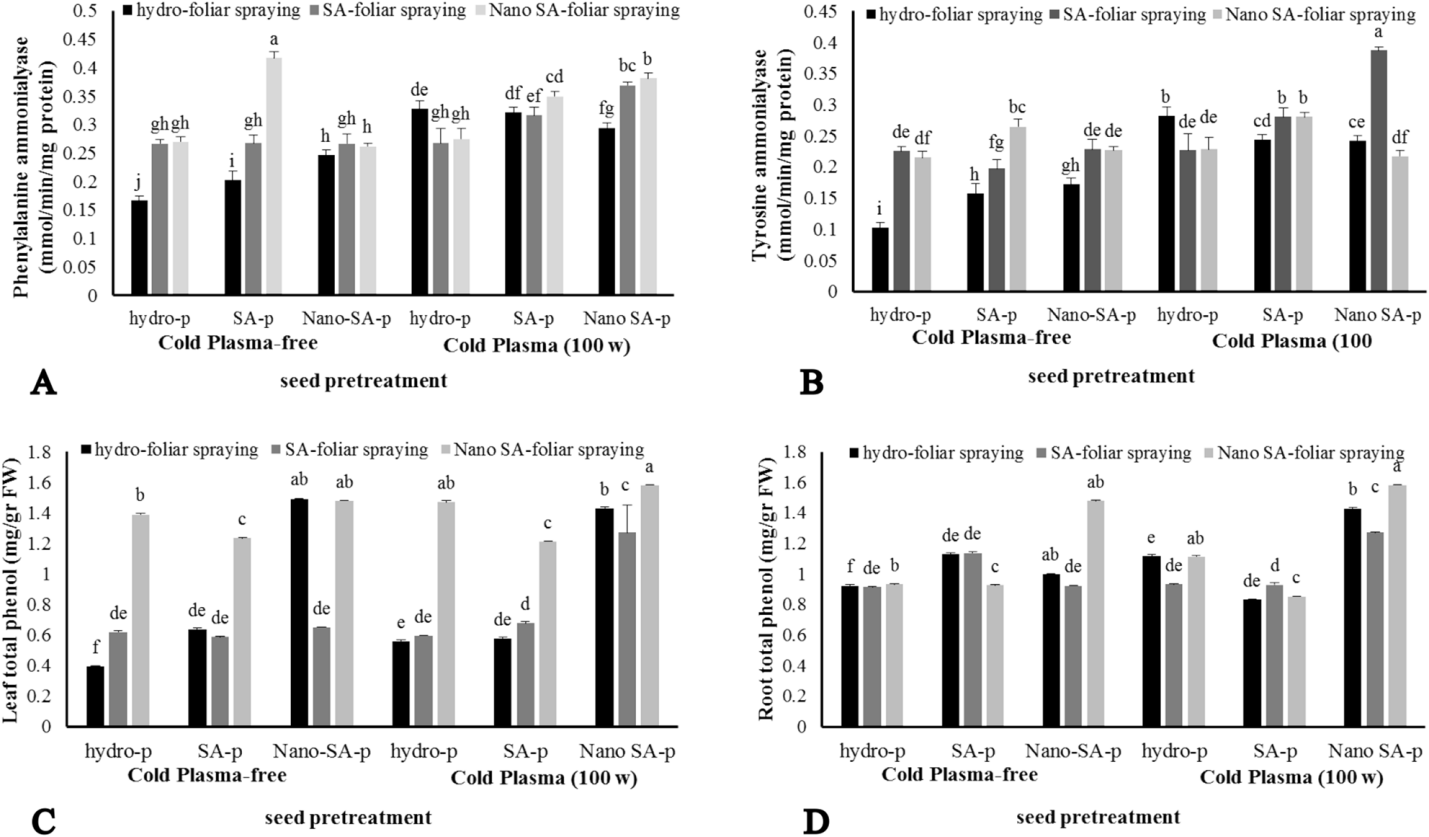
The effect of seed treatment with cold plasma and nano-salicylic acid and foliar spraying of nano-salicylic acid on phenylalanine ammonia-lyase (**A**), tyrosine ammonia-lyase (**B**), and leaf and root total phenol (**C**, **D**) of *S. leriifolia* plant. Values represent mean ± S.E. Different letters indicate significant differences at the 1% level according to the LSD test.

Enhanced PAL activity was observed using CP, both form of SA priming, and both form of SA foliar spraying in *S. leriifolia* plants (P ≤ 0.01) (Fig. 4A). Also, the highest PAL activity was equal to 0.418 mmol/min/mg protein in the CP-free + SA-priming + nano-SA foliar spraying treatment. The activity of this enzyme in *S. leriifolia* plants significantly increased due to CP compared to the CP-free treatment combinations. The lowest PAL activity was observed as 0.167 mmol/min/mg protein in the control (Fig. 4A).

Similar to the results of CP, SA-priming, and SA-foliar spraying effects on PAL activity, TAL activity in *S. leriifolia* plants also significantly (P ≤ 0.01) increased when CP was combined with other treatments. This enzyme's activity rose by 370%, increasing from the control plants' minimum of 0.103 mmol/min/g protein to the highest, which reached 0.387 mmol/min/g protein in the CP + nano-SA priming + SA foliar spray treatment (Fig. 4B). The two forms of SA foliar spraying (SA and nano-SA) exhibited similar TAL activity in the majority of treatment combinations (Fig. 4B).

### Total phenol

The results regarding the effects of CP, SA priming and SA foliar spraying on the total phenol content of *S. leriifolia* leaves and roots are presented in Fig. 4C and D (P ≤ 0.01). It appears that CP and SA priming have a lesser effect on the changes in total phenol content, whereas nano-SA foliar spraying significantly influenced this biochemical trait. The highest total phenol content in the leaves was observed in four treatment combinations : CP-free + nano-SA priming + hydro-foliar spraying, CP-free + nano-SA priming + nano SA-foliar spraying, CP + Hydro priming + nano-SA foliar spraying, and CP + nano-SA priming + nano SA-foliar spraying (Fig. 4C). Total phenol content increased by more than four times in these treatment combinations compared to the control (CP-free + hydro priming + hydro foliar spraying). Additionally, the lowest content of total phenol in the roots and leaves was observed in the control (Fig. 4C and D).

### Secondary metabolites content

**Figure 5:**
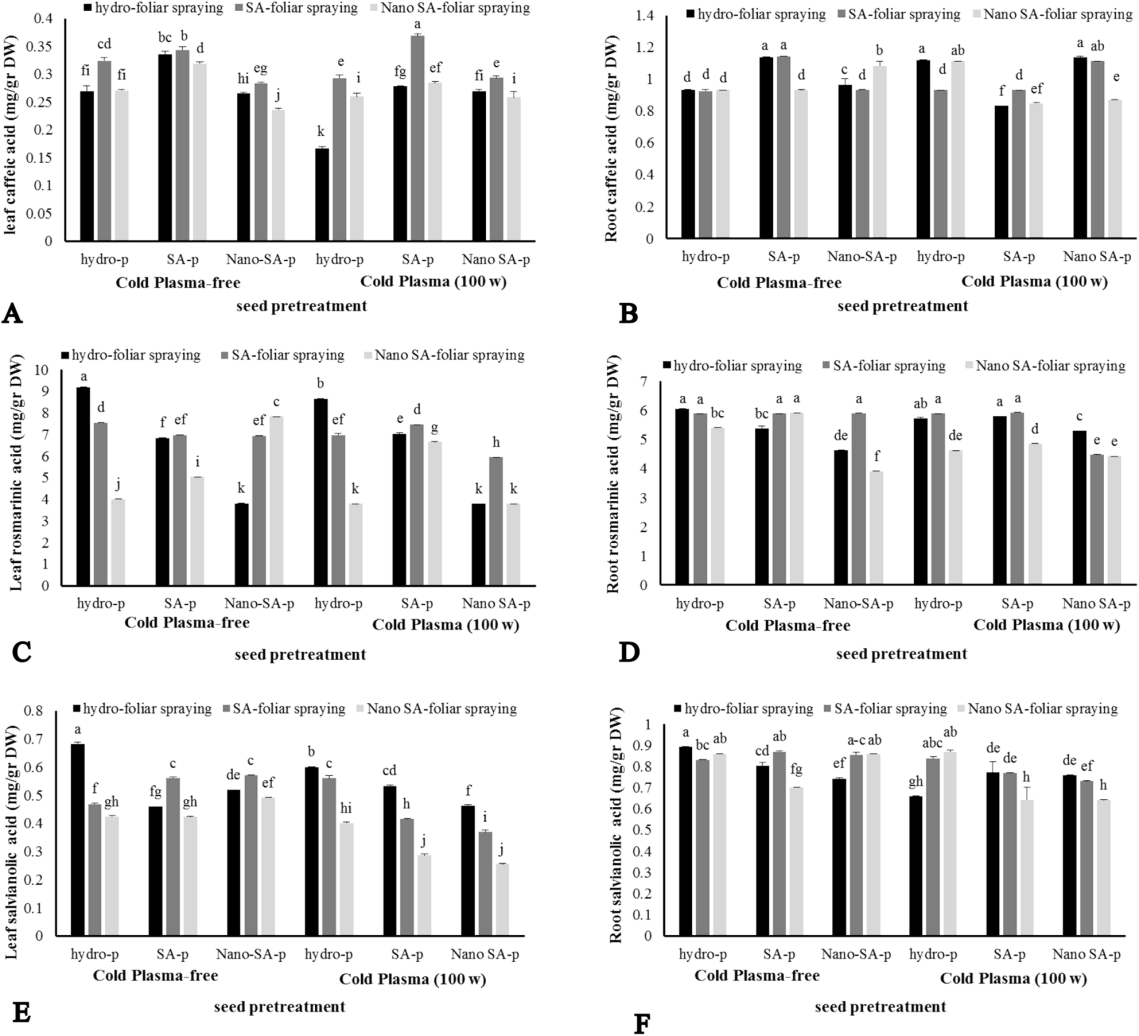
The effect of seed treatment with cold plasma and nano-salicylic acid and foliar spraying of nano- salicylic acid on caffeic acid (**A**, **B**), rosemarinic acid (**C**, **D**), and salvianolic acid (**E**, **F**) contents of leaf and root of *S. leriifolia* plant. Values represent mean ± S.E. Different letters indicate significant differences at the 1% level according to the LSD test.

Changes in the content of rosmarinic acid, caffeic acid, and salvianolic acid in the roots and leaves of *S. leriifolia* had a similar trend (P ≤ 0.01) (Fig. 5A–F). The results of the effect of CP, priming, and foliar spraying of SA on the content of caffeic acid in *S. leriifolia* showed that the maximum amount of caffeic acid was obtained as 0.369 mg g^−1^ DW by CP + priming SA + foliar spraying of SA (Fig. 5A). However, CP and foliar spraying of Nano-SA diminished the biosynthesis of this secondary metabolite. of this secondary metabolite (Fig. 5A,B). Using normal SA priming had an improving effect on caffeic acid content (Fig. 5A).

Figure 5C and D represents the results of rosmarinic acid content under CP, SA-priming, and SA foliar spraying in the leaves and roots of *S. leriifolia*. The changes in the content of this secondary metabolite under the influence of CP, SA priming and foliar spraying-SA mirrored the trends seen for the content of caffeic acid. The highest concentration of rosmarinic acid, 46 mg g^−1^ DW, was observed in control plants. Nano-SA foliar spraying further reduced the presence of this secondary metabolite in most treatment combinations (Fig. 5C).

The decrease in salvianolic acid B content using CP in all treatment combinations was more pronounced than in CP-free treatments (Fig. 5E,F). The most significant reduction of this secondary metabolite was recorded in nano-SA foliar spraying. Consequently, the highest and lowest content of salvianolic acid B were measured as 68.2 and 25.6 mg g^−1^ DW in the control (CP-free, hydro priming, and hydro foliar spraying) and CP + nano-SA priming + nano-SA foliar spraying plants, respectively (Fig. 5E). Moreover, there was no significant difference in the salvianolic acid B content between plants treated with CP + any priming + nano-SA foliar spraying. Priming and foliar spraying of SA (normal and nano form) had a negative effect on salvianolic acid B, which differs from other scientific reports (Fig. 5E).

## Discussion

CP and nano-SA, possessing the aforementioned characteristics, were evaluated to assess their potential in enhancing the growth and phenolic compound production in *S. leriifolia*. The method employed for preparing nano-SA in this experiment revealed that the nanoparticle size was nearly half that of normal particles, signifying the effectiveness of this preparation method. Moradi et al.^32^ previously reported the preparation of zeolite nanoparticles and silica nanoparticles using this method. Considering the results obtained, along with the simplicity and cost-effectiveness of the process and ability to produce a substantial quantity of nanomaterials at room temperature in a short period, this method proves to be effective and practical for nano-SA preparation.

In this experiment, exposing *S. leriifolia* seeds to CP under low-pressure conditions resulted in significant and uneven etching on the uncoated seed surfaces. This observation aligns with previous studies that reported CP exposure causing etching on the seed surfaces of barley and pea seeds^33,34^. This disruption of the regular mesh-like structure can be attributed to the generation of various reactive excited species, such as N_2_^+^, N_2_, OH, O, and O^+^ (Fig. 2C,D). Differences in the energy distribution of the CP may also have played a role^33,35^.

Significant changes in height during the initial growth of the *S. leriifolia* plants were observed upon exposure to CP, and foliar spraying of nano or normal SA, with particularly notable effects from nano-SA foliar spraying. Our findings align with those of Sheteiwy et al.^36^, who demonstrated that CP-treated seeds, either alone or combined with SA priming, significantly increased root and shoot length, dry weight, photosynthetic pigments, and overall growth in *Oryza sativa* seedlings. Zahoranová et al.^37^ similarly reported a noticeable increase in both root and shoot length in maize seeds treated with CP. Typically, the plant cell wall acts as the first barrier against external substances, limiting the effectiveness of applied treatments. Nanoscale materials can easily permeate through the cell wall and reach the plasma membrane. It is evident that the smaller the size of SA particles inside the plant, the more effectively they can participation in various metabolic processes^19^.

Most of the evaluated morphological traits indicated that combining seed priming with both forms of SA and cold plasma had a more pronounced effect compared to the application of cold plasma alone. This positive impact of CP on the growth features may have arisen from significant changes in the biosynthesis of auxins, and cytokinins, as well as their ratio. Additionally, it led to an increase in the content of GA3, and a reduction in ABA levels, which are early biochemical responses to CP, ultimately enhancing the later growth of plants^38,39^. Complex chirality plays a crucial role in how plant attributes and nanomaterial properties, including concentration, chemistry, surface features, size, and shape, interact within plant cell responses^40^. Converting this plant growth regulator into nanoparticles not only increases its surface area but also likely enhances its penetration into the plant. Furthermore, studies involving iron-carbon nanoparticles on pumpkins have confirmed that the substance penetration increases with the decreasing particle size^41^. It appears that foliar spraying of both forms of SA (normal and nano) had a role in leaf length enhancement, with nano-SA showing a more pronounced effect. Extensive research on the effects of SA on plant growth has demonstrated its role in promoting growth through the regulation of cell division and expansion^42–45^, chlorophyll content, and hormonal changes^46^. SA interacts with various other plant growth regulators (PGR), including auxin, ethylene, and gibberellin, to control these cellular processes^47^.

Most SA interactions are linked to auxin^48–51^, with SA potentially influencing auxin transit and accumulation, thereby affecting root growth. Consequently, SA induces the expression of various auxin biosynthetic enzymes, such as TRP aminotransferase of Arabidopsis 1 (TAA1), and the auxin efflux protein PIN1^52^

The current study confirms that the interaction between CP and SA priming, as well as foliar SA spraying, is influenced by the form of SA. Nano-SA was found to enhance certain morphological properties, including plant height, leaf length, and plant fresh weight, more effectively than normal SA. The observed increase in leaf length resulting from SA foliar spraying can be attributed to both an increase in the number of ladder parenchyma cells and a reduction in intercellular space within this tissue^53^. A previous study was suggested that CP seed pretreatment improved the uptake of nitrogen and phosphorus, leading to increases growth and leaf area^54^. However, contradictory results have been reported with cold plasma seed pretreatment^54^, likely due to variations in genotype, plant species, plasma concentration, treatment duration, gas used, and differing environmental conditions for each experiment^55^.

Nonetheless, *S. leriifolia* exhibited mixed responses to the various combinations of three factors. Notably, an increasing trend in fresh and dry weight was obtained using nano-SA compared to normal SA. In a similar context, SA seed pretreatment in black cumin^56^ and foliar spraying in chickpeas^27^ and corn plants^57^ enhanced both fresh and dry weight. The impact of SA on plant growth is dependent on various factors, including, SA concentration, application method and timing, plant species, and growth stage^58,59^.

CP enhances dry weight by increasing the amount of transfer reserves, depletion percentage, and seed storage efficiency^60^. Simultaneously, CP modifies the seed coat, increasing water and gas exchange and resulting in improved seed permeability to SA. Interestingly, some treatment combinations had no significant effect on the content of both chlorophylls compared to the control, and in certain combinations, their content even decreased. In contrast to our results, Nemati Mirak et al.^30^ reported a significant effect of both forms of SA on chlorophyll content. Furthermore, CP improved the growth of okra seedlings (*Abelmoschus esculentus* L.), and wheat by enhancing the photosynthetic pigments^61,62^.

CP, in combination with both forms of SA priming and foliar spraying, stimulated the activity of PAL and TAL enzymes in *S. leriifolia* compared to the control, with a notable increase observed in PAL enzyme activity. This observation aligns with the findings of other researchers who reported increased PAL activity in wheat under CP treatment, as well as under CP and SA treatments alone, or in combination in *Oryza sativa*^36,63^. Phenolic compounds in *S. leriifolia* plants increased when subjected to different combinations of the three treatments compared to the control, with nano-SA spraying showing an impressive threefold increase.. The positive impact of CP and SA on increasing total phenols in *Echinacea purpurea* and *Lavandula angustifolia*, respectively, has been previously documented^16^, which is in line with the results described in the present study. It is likely that nano-SA is more effectively absorbed and participates in various metabolic processes^19^. One facet of plant defense responses involves the increased production of phenolic compounds, facilitated by the rapid expression and activity of the PAL gene. PAL and TAL enzymes are recognized as primary participants in the biosynthesis of phenolic compounds, producing t-cinnamic acid and p-coumaric acid, respectively, which serve as precursors in the phenolic compound pathway^64^. Based on our research supports the findings of Berner and Westhuizen^65^, emphasizing the significant role of the PAL enzyme in defense responses, ultimately leading to the biosynthesis of bioactive compounds, including phenolic compounds. The various combinations of CP and SA were found to have a detrimental effect on the quantities of secondary metabolites, particularly rosmarinic acid and salvianolic acid. Most of these combinations resulted in a decrease in the production of these secondary metabolites, in contrast to the results observed for PAL and TAL activity, as well as phenol content. Among the treatments in this experiment, the reduction in the quantity of caffeic acid content was comparatively lower than the reductions observed in the amounts of rosmarinic acid and salvianolic acid. It's worth noting that salvianolic acid biosynthesis occurs through the conversion of rosmarinic acid, during the biosynthesis of these secondary metabolites. Therefore, the reduction in rosmarinic acid under the treatments may have led to a decrease in salvianolic acid (Fig. 1). Several factors could contribute to these results: First, variations in growth conditions and plant growth stages may have played a role^28^. Second, the plant might have allocated its energy towards improving certain morphological traits in the post-treatment period. Third, despite enzyme activation, it is possible that the precursors needed for the biosynthesis of these secondary metabolites have not yet had a chance to be converted. Moreover, the plant might be directing its resources toward the biosynthesis of other phenolic compounds.

## Conclusion

In general, SEM images revealed that CP could significantly alter the regular surface structure of *S. leriifolia* seeds, and the morphological and biochemical attributes of immature plants were notably enhanced when using nano-SA in comparison to normal SA. The total phenol content increased as a result of these applications, but their impact on the biosynthesis of specific phenolic acids such as rosmarinic acid, caffeic acid, and Salvianolic acid B during the initial growth stages of the plants was limited. The application of CP during seed germination and nano-SA during the plant growth stage demonstrated potential dual benefits. These applications triggered enzyme activity involved in phenolic acid biosynthesis and enhanced growth pathways, which may collectively have a positive effect on secondary metabolite biosynthesis in mature plants. Therefore, further research, considering gene expression related to the secondary metabolite biosynthesis pathway and the involved signaling cascades, is recommended to optimize the growth of *S. leriifolia* plants and the biosynthesis of their secondary metabolites in field conditions using CP, and nano-SA as environmentally friendly methods.

## Materials and methods

### Seed preparation

The seeds of *S. leriifolia* were collected from their natural habitats in Bajestan, located in Khorasan, Iran, in early June (34.31 N, 58.10 E). A plant taxonomist, in accordance with institutional, national, and international guidelines, and regulations, verified the collected seeds. Subsequently, they were transported to the Tehran pest and disease of plant Research Institute (PPDRI) and stored at 4 °C.

### Nano salicylic acid preparation and particle size

A high-energy Planetary ball mill (Amin Asia Fanavar Pars, Iran) was used to grind the SA particles at a speed of 300 rpm for one hour, resulting in finely ground SA particles. The SA used in this experiment was obtained from Sigma-Aldrich, Germany. The morphology of the nano-SA particles was examined and confirmed using Field Emission Scanning Electron Microscopy MIRA3 TESCAN (Brno, Czech Republic). The particle size of the nano-SA was also determined using FESEM on a MIRA3 TESCAN instrument.

### Cold plasma, nano-salicylic acid priming, and nano-salicylic acid foliar spraying

The seeds of *S. leriifolia* have hard coats. Based on the results of the preliminary experiment, uncoated seeds were used in this study. CP (in two power levels, including CP-free, and 100 w for 4 mins), and SA priming (three concentrations including hydro priming, 2 mM of the normal size of SA, and nano-SA), were used to treat un-coated seeds. To create FESEM images of the treated seeds, a standard base was attached with glume on both sides, and a conductive layer with a diameter of 125 nm gold (Au) was applied to the seeds before starting photography at 700 × magnification using (MIRA3 TESCAN (Brno, Czech Republic).

### Seed culture on the farm

After germination, the treated seeds were cultivated in the farm and subjected to foliar spraying with SA at the six-leaf stage, with three different concentrations including hydro, 2 mM of the normal size of SA, and nano-SA. For land preparation, the soil was semi-deep-ploughed in the fall and disked before sowing the seeds. The chemical and physical properties of the experimental field soil are presented in Table 1. Each treatment plot in this experiment covered an area of 12 square meters, comprising five rows with eight plants in each row. The spacing between plants within a row was 40 cm, and there was a one-meter gap between different treatments. Weed control was consistently carried out. The treated seeds were sown on the farm on March 16, 2017, at a depth of 1.5–1 cm. Foliar spraying with SA treatments was performed using a hand sprayer until all leaf surfaces of the plants were thoroughly wetted. After one week, plants were collected for analysis of the properties listed below.

**Table 1:** The chemical properties of farm soil.

	pH	EC (ds/m)	B (ppm)	Mn (ppm)	Cu (ppm)	Zn (ppm)	Fe (ppm)	P (ppm)	K (ppm)	N%	TNV	OC
Soil characteristics	7.77	2.16	1.32	7.23	1.69	0.85	5.11	35	396.6	0.22	22.07	2.26

The physical properties of farm soil												
	Testure	Sand%	Silt%	Clay%								
Soil characteristics	Loamy sandy	56	28	16								

### Plant height and leaf length

A metric ruler was used to measure height and leaf length of plants.

### Measurement of chlorophyll a, and b

The chlorophyll contents (a and b) were measured using the Arnon method ^66^. To prepare the samples, 0.5 g of the fresh plant material was ground with liquid nitrogen, and then 20 ml of 80% acetone was added. The samples were subsequently vortexed and centrifuged for 10 min at 6000 rpm. The resulting supernatants were transferred to new tubes. Absorbance measurements of chlorophyll a and b were taken at 663 nm and 645 nm, respectively, using a Unico UV–Vis spectrophotometer, (2150, China). The concentrations of chlorophyll a and b were calculated in milligrams per gram of fresh sample weight using the formula 1 and 2.

1 $$\mathrm{Chlorophyll \, a }(19.3*\mathrm{A}663-0/86*\mathrm{A}645)\, \mathrm{ v}/100 \, \mathrm{w}$$

2 $$\mathrm{Chlorophyll \, b }(19.3*\mathrm{A}645-3/6*\mathrm{A}663) \, \mathrm{ v}/100\, \mathrm{w}$$

(V) Volume of solution, (A) absorption at 663 and 645 nm, (W) the fresh weight of the sample (g).

### Plant fresh and dry weight

The fresh and dry weight of the plant were measured using a scale with an accuracy of 0.001 (digital Sartorius model CP 423S). To measure the dry weight, an oven (Tara Teb Company) set at a temperature of 70 °C for 48 h was used.

### Activity of phenylalanine ammonia-lyase, and tyrosine ammonia-lyase

The activity of PAL and TAL enzymes was measured using the method described by Beaudoin-Eagan and Thorpe method^64^. Spectrophotometer measurements of the specific enzyme activity were based on the generation of kinematic trans-acid for PAL at 290 nm, and coumaric acid for tyrosine ammonia at 333 nm (UV-2100).

### Total phenol

The total phenolic content was determined using the Folin-Ciocalteu method^67^.

### Phenolic acids measurement

To quantify phenolic acids, including caffeic acid, rosmarinic acid, and salvianolic acid B, we employed an extraction method described by Wang et al.^68^, followed by HPLC analysis using a Waters Alliance model 2695 with repeated three times (n = 3).

### Statistical analysis

The experiment in this study was designed as a randomized complete block in a factorial arrangement, with three replications, and was conducted at an agricultural farm in Tehran province (latitude 35.75, longitude 51.375, and altitude 1333 above sea level). Each treatment involved the cultivation of 40 plants. The gathered data was analyzed using SAS software version 9.4 (SAS Institute, Cary, NC, USA) through analysis of variance (ANOVA). The significant differences between treatments were determined using LSD multiple-range tests (p < 0.05).

## Data Availability

The datasets used and/or analyzed during the current study are available from the corresponding author on reasonable request.
